# Impact of Nerve-Sparing Techniques on Prostate-Specific Antigen Persistence Following Robot-Assisted Radical Prostatectomy: A Multivariable Analysis of Clinical and Pathological Predictors

**DOI:** 10.3390/diagnostics15080987

**Published:** 2025-04-13

**Authors:** Lorenzo Spirito, Carmine Sciorio, Lorenzo Romano, Antonio Di Girolamo, Antonio Ruffo, Giuseppe Romeo, Felice Crocetto, Luigi Napolitano, Marco Stizzo, Francesco Bottone, Carmelo Quattrone, Vittorio Imperatore

**Affiliations:** 1 Unit of Urology, Department of Woman, Child and General and Specialized Surgery, University of Campania “Luigi Vanvitelli”, 80131 Naples, Italy; lorenzo.romano@unicampania.it (L.R.); marcostizzo@hotmail.com (M.S.); carmeloquattrone@hotmail.it (C.Q.); 2 UOC Urologia Ospedale Manzoni, 23900 Lecco, Italy; carmine.sciorio@gmail.com; 3 AORN Moscati di Avellino, UOC di Urologia, 83100 Avellino, Italy; antonio.digirolamo@hotmail.it (A.D.G.); v.imperatore1@gmail.com (V.I.); 4 Dipartimento di Medicina e di Scienze della Salute “Vincenzo Tiberio”, 86100 Campobasso, Italy; antonio.ruffo7@gmail.com; 5 Ospedale AORN “A.Cardarelli”, 80131 Napoli, Italy; gp.romeo@icloud.com; 6 Department of Neurosciences, Science of Reproduction and Odontostomatology, University of Naples Federico II, 80131 Naples, Italy; felice.crocetto@unina.it; 7 Azienda Sanitaria Locale (ASL) Salerno, via Vernieri, 84125 Salerno, Italy; nluigi89@libero.it; 8 DAI Medico Chirurgico di Alta Specialità, UOC Urologia Università degli Studi della Campania “Luigi Vanvitelli”, 80138 Napoli, Italy; bottonefrancesco@yahoo.it

**Keywords:** prostate cancer, radical prostatectomy, robot-assisted surgery, nerve-sparing, PSA

## Abstract

**Background/Objectives:** Prostate-specific antigen (PSA) persistence, defined as a postoperative PSA level ≥ 0.1 ng/mL measured within 4–8 weeks after radical prostatectomy (RP), predicts biochemical recurrence (BCR) and adverse oncological outcomes. The influence of nerve-sparing (NS) surgical techniques on PSA persistence remains debated, especially among patients with high-risk pathological features. This study aimed to evaluate the impact of NS techniques on PSA persistence following robot-assisted radical prostatectomy (RARP), considering tumor characteristics, surgical parameters, and patient-specific factors. **Methods:** A retrospective cohort analysis was performed on 779 patients who underwent RARP at a single institution between January 2002 and December 2015. The inclusion criteria consisted of histologically confirmed prostate cancer with available preoperative and postoperative data, including PSA measurements taken 4–8 weeks after surgery. PSA persistence served as the primary outcome. Statistical analyses included descriptive statistics, univariate and multivariable logistic regression models to identify predictors of PSA persistence, and Spearman’s correlation along with the Kruskal–Wallis H test to evaluate associations. **Results:** Of the 779 patients included, 55% underwent NS surgery (51% unilateral, 49% bilateral). The mean preoperative PSA was 11.85 ng/mL (SD: 7.63), while the mean postoperative PSA was 0.70 ng/mL (SD: 4.42). An elevated postoperative PSA was associated with a larger tumor size (r = 0.1285, *p* < 0.001), advanced pathological stages (χ^2^ = 45.10, *p* = 3.79 × 10^−9^), and higher Gleason scores (χ^2^ = 24.74, *p* = 1.57 × 10^−4^). NS surgery correlated with a lower postoperative PSA (mean: 0.20 ng/mL) compared to non-NS procedures (mean: 0.65 ng/mL), with slight differences between unilateral (mean: 0.30 ng/mL) and bilateral (mean: 0.35 ng/mL) NS approaches. Multivariable regression analysis identified advanced pathological stage (coefficient = 1.16, *p* = 0.04) as an independent predictor of PSA persistence, while NS techniques had no significant independent effect (coefficient = −0.01, *p* = 0.99). **Conclusions:** Nerve-sparing surgical techniques do not independently predict PSA persistence after RARP when adjusting for tumor-related factors and confounders. Advanced pathological stage, particularly stage pT3b, primarily determines PSA persistence. These findings highlight the necessity of personalized surgical planning informed by preoperative imaging and patient-centered decision making to optimize oncological and functional outcomes.

## 1. Introduction

Prostate cancer (PCa) is the most commonly diagnosed malignancy among men globally [1], with a significant impact on both morbidity and mortality [2,3]. Radical prostatectomy (RP) is the standard treatment for localized PCa, offering excellent long-term cancer control. However, postoperative prostate-specific antigen (PSA) levels remain a key biomarker for assessing surgical outcomes. Persistent PSA, defined as a PSA level of ≥0.1 ng/mL measured 4–8 weeks after surgery, is a significant predictor of biochemical recurrence (BCR) and poor oncological prognosis [4]. Addressing the factors associated with PSA persistence is essential for improving surgical planning, guiding adjuvant therapy, and informing patient management [5,6].

Established predictors of biochemical persistence (BP) include high preoperative PSA levels, advanced Gleason scores, and pathological tumor stage [7]. Furthermore, emerging evidence highlights correlations between prostate volume, body mass index (BMI), and surgical outcomes [8].

The role of surgical techniques, particularly nerve-sparing (NS) approaches, in PSA persistence remains controversial. NS surgery aims to preserve erectile function by sparing neurovascular bundles. Still, it raises concerns regarding increased rates of positive surgical margins (PSMs), particularly in high-risk patients with extracapsular extension (ECE) or seminal vesicle invasion [9]. Despite such concerns, recent advancements in imaging modalities, including multiparametric MRI (mpMRI) [10], have improved preoperative risk stratification and surgical planning. Studies indicate that lesion localization via mpMRI significantly enhances prostate cancer detection rates in biopsy-naïve patients [11]. Also, emerging interdisciplinary perspectives underscore potential systemic factors influencing PCa outcomes, such as the interplay between prostate and gut microbiota [12].

Persistent PSA levels after RP present a dual challenge [13]: the risk of progression to BCR and the trade-off between oncological control and functional preservation. Biochemical persistence (BP) [14] reflects the presence of residual microscopic or macroscopic disease. Previous studies have identified critical factors influencing BP, including preoperative PSA levels, Gleason scores, pathological tumor stage, and lymph node involvement [7]. Despite these findings, considerable heterogeneity persists regarding the role of surgical techniques, particularly nerve-sparing (NS) surgery [15,16], in PSA persistence. NS techniques aim to preserve erectile function by sparing the neurovascular bundles but may compromise oncological outcomes in patients with high-risk diseases [17]. While some studies suggest that NS techniques increase the likelihood of residual disease, others report no significant impact on oncological outcomes when performed in appropriately selected patients [18]. Evaluating the effect of nerve-sparing (NS) on outcomes is made difficult by the use of adjuvant radiotherapy [19], particularly within advanced pathological stages [20,21]. Simeone et al. [22] reported that NS techniques may leave microscopic residual disease near spared bundles, contributing to PSA persistence. However, other studies suggest that NS surgery, when performed in carefully selected patients, does not compromise oncological outcomes and may even reduce BP rates by preserving critical structures and minimizing surgical trauma [23,24]. Balancing oncological and functional outcomes is therefore critical in surgical decision making, particularly as patients increasingly prioritize the quality of life after RP [25]. Also, high-volume surgeons are more likely to achieve optimal oncological and functional outcomes, even in patients undergoing NS surgery [26]. Finally, advanced imaging modalities and molecular biomarkers are also being explored as tools for preoperative risk stratification, enabling personalized surgical approaches [27].

This study evaluates the impact of nerve-sparing (NS) techniques on PSA persistence after robot-assisted radical prostatectomy, with attention to confounding factors like tumor pathology and lymph node involvement. It explores the relationship between tumor characteristics such as Gleason score, pathological stage, and lymph node status and their influence on PSA persistence. Additionally, it examines whether NS techniques have an independent effect on PSA persistence when accounting for tumor-related variables. The findings aim to inform evidence-based recommendations for surgical planning, particularly regarding the suitability of NS approaches for patients with high-risk diseases. This study addresses a critical gap in the understanding of PSA persistence after RARP, particularly the role of NS techniques in influencing oncological outcomes [28]. By evaluating clinical, pathological, and surgical factors in a comprehensive multivariable framework, the findings aim to provide actionable insights for optimizing prostate cancer management. The study underscores the importance of individualized surgical planning, robust preoperative risk stratification, and multidisciplinary decision making to achieve the dual goals of oncological control and functional preservation [29,30].

## 2. Patients and Methods

### 2.1. Study Population

This retrospective cohort study analyzed data from patients undergoing RARP at a single institution between January 2002 and December 2015. The inclusion criteria included histologically confirmed prostate cancer, available preoperative and postoperative clinical data, and PSA levels measured 4–8 weeks postoperatively. Patients were excluded if they received adjuvant radiotherapy or androgen deprivation therapy within the first eight weeks post-surgery, as these interventions could confound PSA persistence. Additional exclusion criteria included incomplete clinical datasets and a history of prior prostate surgeries.

### 2.2. Variables Considered

This study collected data on preoperative variables, including age, preoperative PSA levels, Gleason total preoperative score, prostate weight, and tumor length. Postoperative pathological data included Gleason’s total postoperative score, pathological stage (pT), and lymph node status (number of positive and total nodes sampled). The surgical factors considered were nerve-sparing techniques (categorized as bilateral, unilateral, or non-nerve-sparing) and the side on which nerve-sparing was performed.

The primary outcome was biochemical persistence, defined as a PSA level ≥0.1 ng/mL measured 4–8 weeks postoperatively. The secondary outcomes included associations between nerve-sparing and positive surgical margins.

### 2.3. Statistical Analysis

Descriptive statistics were used to summarize patient demographics and clinical variables. Continuous variables, such as preoperative PSA and prostate weight, were expressed as medians and interquartile ranges (IQRs), while categorical variables, such as pathological stage and nerve-sparing status, were presented as frequencies and percentages. Univariate analyses were performed to explore the relationships between clinical and pathological predictors with postoperative PSA levels. Spearman’s correlation was used for continuous predictors, and the Kruskal–Wallis H test was employed for categorical variables.

Multivariable logistic regression analysis was conducted to identify independent predictors of PSA persistence, adjusting for potential confounders such as pathological stage, lymph node positivity, and positive surgical margins. Model performance was evaluated using the area under the receiver operating characteristic (ROC) curve. Statistical significance was defined as *p* < 0.05, and all analyses were performed using R software (4.4.0).

This methodological framework aims to disentangle the impact of nerve-sparing techniques on PSA persistence while accounting for confounding factors, ensuring robust and clinically meaningful results.

## 3. Results

### 3.1. Descriptive Statistics

A total of 779 patients were included in this retrospective cohort study. Of these, 55% (428 patients) underwent nerve-sparing (NS) surgery, while the remaining 45% (351 patients) underwent non-nerve-sparing (non-NS) procedures. Among the NS group, 51% (219 patients) had unilateral nerve-sparing, and 49% (209 patients) had bilateral nerve-sparing. The average preoperative PSA level was 11.85 ng/mL, with a standard deviation of 7.63, ranging from 2.00 to 34.00 ng/mL. Patients in this study had a mean age of 63.46 years (standard deviation: 6.48 years; range: 43–85 years). Tumor characteristics included an average tumor length of 17.47 mm (standard deviation: 21.43 mm), and the mean weight of the prostate gland was 42.17 g (standard deviation: 22.98 g). The average postoperative PSA level was 0.70 ng/mL, though it exhibited a wide variation, with a standard deviation of 4.42 and a range of 0.01 to 79.00 ng/mL.

In terms of surgical factors, nerve-sparing techniques were utilized in 55% of cases. Of these, 51% involved unilateral nerve-sparing, while the remainder were bilateral or non-nerve-sparing procedures. These data underlined the variability in surgical approaches and patient characteristics within the cohort (Table 1).

### 3.2. Univariate Analysis of Clinical and Surgical Factors and Postoperative PSA Levels

The univariate analyses revealed several noteworthy associations between postoperative PSA levels and the clinical or surgical variables under consideration. As shown in Table 2, neither preoperative PSA (r = −0.0487, *p* = 0.1886) nor patient age (r = −0.0086, *p* = 0.8163) significantly correlated with the postoperative PSA. In contrast, tumor length (r = 0.1285, *p* < 0.001) and total lymph nodes examined (r = 0.1298, *p* = 0.012) displayed positive correlations, suggesting that larger tumors and more extensive lymph node dissections may contribute to an elevated postoperative PSA. Findings from the Kruskal–Wallis H test (Table 3) further indicated that higher Gleason scores (preoperative: χ^2^ = 24.2127, *p* = 4.77 × 10^−4^; postoperative: χ^2^ = 24.7385, *p* = 1.57 × 10^−4^) and advanced tumor stage (χ^2^ = 45.1013, *p* = 3.79 × 10^−9^) were each associated with elevated PSA levels, reflecting more aggressive disease. Additionally, nerve-sparing surgery (χ^2^ = 3.8608, *p* = 0.0494) and the side of nerve-sparing (χ^2^ = 6.5022, *p* = 0.0387) emerged as significant factors, showing lower PSA levels in nerve-sparing cohorts, particularly for unilateral sparing. Table 4 corroborated these results: mean PSA values were progressively higher in subgroups with more advanced Gleason scores (e.g., Gleason > 7: mean = 4.50 ng/mL) and disease stages (e.g., pT3b: mean = 2.14 ng/mL). Patients undergoing nerve-sparing surgery exhibited a lower mean PSA (0.20 ng/mL) compared to those without nerve-sparing (0.65 ng/mL), with only modest differences between unilateral (0.30 ng/mL) and bilateral (0.35 ng/mL) approaches. Overall, these univariate findings underscore the influence of disease aggressiveness, surgical strategy, and pathological staging on postoperative PSA.

### 3.3. Multivariable Logistic Regression Analysis

**Figure 1:**
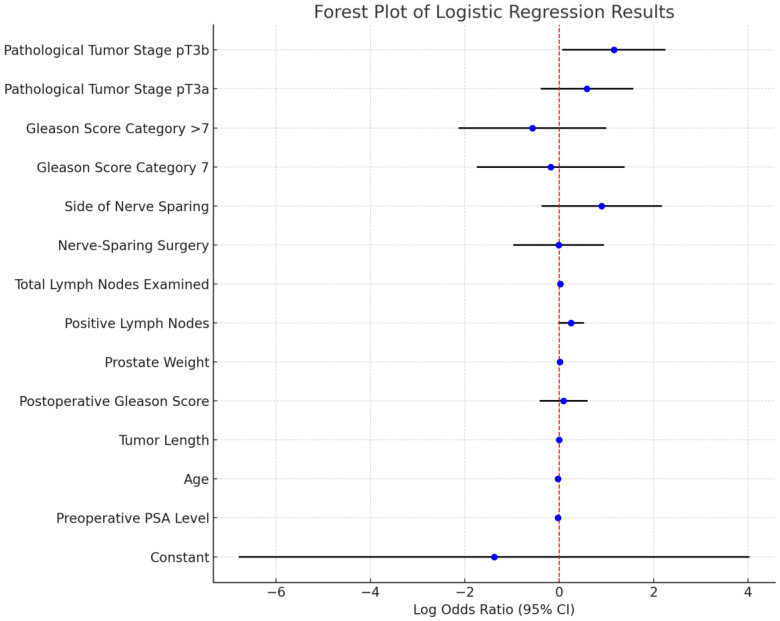
Forest plot of logistic regression results.

Subsequent multivariable logistic regression offered an additional perspective on predictors of PSA persistence (Table 5 and Figure 1). While most variables did not achieve formal statistical significance, lymph node positivity showed a near-significant trend (coefficient = 0.25, *p* = 0.08), suggesting a potential role for nodal disease in driving PSA persistence. Pathological tumor stage emerged as a key factor, with advanced disease (pT3b stage, involving seminal vesicle invasion) strongly associated with residual PSA (coefficient = 1.16, *p* = 0.04), potentially reflecting microscopic disease beyond the surgical margin. Other variables, such as preoperative PSA (coefficient = −0.03, *p* = 0.33), tumor length, Gleason score (coefficient = 0.09, *p* = 0.74), and prostate weight, did not appear to be significant predictors of early PSA persistence. Likewise, nerve-sparing surgery—whether unilateral or bilateral—showed no meaningful association (coefficient = −0.01, *p* = 0.99), nor did the side of nerve-sparing (coefficient = 0.90, *p* = 0.16). In sum, although the advanced pathological stage proved to be a critical determinant of PSA persistence, nerve-sparing techniques alone did not appear to influence postoperative PSA outcomes when controlling for confounders. These observations underscore the need for further research into how factors such as surgeon expertise and patient selection may refine the relationship between nerve-sparing and oncologic results.

## 4. Discussion

Postoperative prostate-specific antigen (PSA) measurement is critical in evaluating the effectiveness of radical prostatectomy (RP) for localized prostate cancer (PCa). Studies such as those by Tourinho-Barbosa et al. [31] have elucidated the prognostic implications of PSA persistence, emphasizing its association with aggressive pathological features and poorer outcomes.

In line with these findings, imaging advancements like multiparametric MRI (mpMRI) and prostate-specific membrane antigen (PSMA) PET scans are transforming surgical planning and follow-up care. PSMA PET, in particular, demonstrates heightened sensitivity in detecting micrometastases or residual disease, as highlighted by Houshmand et al. [32]. BRCA germline mutations have been identified as significant contributors to prostate cancer progression, with tailored approaches increasingly advocated to improve management in mutation carriers [33]. Furthermore, advancements in combinatorial methodologies—such as integrating mpMRI with the prostate health index (PHI) using artificial neural networks—have shown promise in improving the identification of clinically significant PCa at diagnosis. As Gentile et al. demonstrated [34], this approach achieved high sensitivity and specificity, suggesting its potential utility in personalizing prostate cancer management [35,36].

Tumor pathology remains a cornerstone in predicting PSA persistence. Higher Gleason scores and adverse pathological stages, such as seminal vesicle invasion or extracapsular extension, significantly elevate the risk of BP. Moreover, systemic factors such as obesity and metabolic syndrome are gaining attention for their role in complicating surgical dissection and increasing the likelihood of positive surgical margins (PSMs). Barone et al. [8] have provided compelling evidence of a correlation between body mass index (BMI) and prostate volume, underscoring the need for tailored preoperative strategies to mitigate these risks.

Emerging evidence also points to the role of systemic health in PCa progression. The COVID-19 pandemic has provided a unique context for understanding these interactions. Di Lorenzo et al. [37] reported on the clinical characteristics of patients with metastatic prostate cancer infected with COVID-19. Their findings highlight the potential for aggressive disease progression in patients who are COVID-19-positive with advanced PCa, especially those undergoing hormonal therapy. Investigating whether hormonal therapy acts as a protective or risk factor in such contexts is crucial for guiding future treatment protocols. Similarly, others have also noted how the SARS-CoV-2 pandemic impacted timely care and could influence oncological outcomes [38]. These findings underline the necessity of uninterrupted and timely care to optimize oncological and functional results.

Imaging techniques have undergone rapid evolution and now play a major role in risk stratification and surgical planning. Multiparametric magnetic resonance imaging (mpMRI) offers detailed visualization of suspicious prostate lesions, their relationship to the surrounding structures, and possible extraprostatic extension [39]. With mpMRI, surgeons can map areas that need more aggressive resection versus zones suitable for nerve-sparing (NS) approaches. Advanced imaging may further help identify high-risk regions near the neurovascular bundles that are responsible for erectile function. Structured reporting, increasingly supported by software-assisted platforms, improves diagnostic consistency by standardizing the way images are interpreted and documented [40].

Nerve-sparing robot-assisted radical prostatectomy, introduced decades ago, seeks to preserve erectile function by sparing the neurovascular bundles that run alongside the prostate. Previous research [22,41] has suggested that positive surgical margins are more common in individuals undergoing NS surgery when high-risk pathological features are present. However, the present study—and others like it—show that, after controlling for tumor pathology and lymph node involvement, nerve-sparing techniques do not necessarily lead to higher rates of persistent PSA. This indicates that careful patient selection, guided by robust imaging data and a thorough pathological assessment, allows a surgeon with expertise in NS techniques to achieve oncological results comparable to more radical, non-nerve-sparing resections.

Non-nerve-sparing procedures, sometimes termed “wide excision” or “extensive resection,” entail removing a broader margin of tissue around the prostate, including more of the periprostatic fascia and neurovascular structures. However, the resultant damage to nerves can substantially affect quality of life, leading to higher rates of erectile dysfunction and sometimes impacting urinary continence [42,43]. One major technological advancement that may serve to detect persistent/recurrent disease after prostatectomy is the use of prostate-specific membrane antigen (PSMA) positron emission tomography (PET) [44]. Additionally, genomic assays—such as Decipher, Oncotype DX, and Prolaris—offer molecular risk stratification that complements imaging findings by identifying tumors at heightened risk of recurrence despite apparently localized disease [45].

While our findings contribute to the ongoing dialog surrounding optimal surgical techniques in prostate cancer, several limitations, inherent to the retrospective design, temper our conclusions. The study’s timeframe, spanning 2002 to 2015, encompasses a period of significant evolution in RARP techniques and perioperative care. Due to retrospective data collection, fully accounting for these advancements and surgeon experience—a recognized determinant of surgical success—was not feasible, precluding us from drawing definitive causal inferences. Our definition of PSA persistence (≥0.1 ng/mL within 4–8 weeks postoperatively) is potentially susceptible to assay variability, a factor which we did not explicitly control for. Furthermore, selection bias likely contributed to the observed discrepancy between univariate and multivariable analyses concerning nerve-sparing, as nerve-sparing procedures are frequently favored in patients presenting with lower-risk disease. The impact of BMI, a potential confounder, was acknowledged but not incorporated into the regression models. Crucially, the absence of detailed data on positive surgical margin (PSM) rates, stratified by nerve-sparing status, hinders a comprehensive assessment of oncologic safety in the context of nerve preservation. We can, however, confirm that any adjuvant therapy was administered after the postoperative PSA assessment, ensuring that this did not influence our persistence data. Ultimately, although limited by the retrospective nature, our analyses indicate that advanced pathological features, rather than the nerve-sparing technique itself, predominantly dictate the risk of PSA persistence.

Moving forward, larger prospective or multicenter studies that implement standardized surgical protocols could offer more definitive insights and enhance the generalizability of the findings. Techniques that integrate advanced imaging (like mpMRI or PSMA PET), standardized pathological grading, and consistent definitions of nerve-sparing strategies would help ensure more comparable data across different institutions. Another important step is the inclusion of longer follow-up times to assess how early PSA persistence translates into long-term clinical outcomes, such as definitive BCR, metastatic progression, or mortality.

Meanwhile, research that delves into the role of obesity, metabolic syndrome, and other potentially modifiable patient factors—such as smoking status or dietary habits—could suggest preoperative interventions that improve surgical margins and reduce PSA persistence [46]. For instance, targeted weight loss, improved nutritional support, or immunomodulatory strategies might optimize the tumor microenvironment or facilitate better surgical resection. Studies examining the prostate–gut microbiome axis might eventually identify specific bacterial signatures correlated with persistent PSA or adverse pathology [12]. If such profiles are shown to be modifiable, it could open the door to novel interventions that shift the microbiome before or after surgery to improve oncological control.

## 5. Conclusions

In conclusion, our findings underscored that advanced pathological features—especially pT3b stage and higher Gleason scores—were the primary drivers of persistent PSA after robot-assisted radical prostatectomy. In contrast, nerve-sparing techniques, whether unilateral or bilateral, did not independently predict PSA persistence once tumor characteristics were accounted for. This suggests that well-selected patients can potentially benefit from preserving neurovascular bundles without compromising oncological safety. Preoperative imaging modalities, such as multiparametric MRI and PSMA PET, can facilitate the precise localization of high-risk disease, allowing surgeons to tailor margins to tumor biology while maintaining erectile function. Looking ahead, standardizing surgical protocols, extending follow-up durations, and incorporating molecular markers could further elucidate the interplay between nerve-sparing and prostate cancer outcomes. Ultimately, a multidisciplinary approach that combines accurate risk stratification with patient-centered decision making is critical to optimizing both oncologic control and functional preservation in prostate cancer management.

## Figures and Tables

**Table 1:** Descriptive statistics of the study population.

Variable	Mean	Standard Deviation	Minimum	Maximum
Preoperative PSA Level	11.85	7.63	2.00	34.00
Age	63.46	6.48	43.00	85.00
Preoperative Gleason Score	7.26	1.08	4.00	10.00
Tumor Length (mm)	17.47	21.43	0.00	205.00
Postoperative Gleason Score	7.42	0.91	5.00	10.00
Prostate Weight (g)	42.17	22.98	15.00	214.00
Total Lymph Nodes Examined	15.68	8.24	1.00	45.00
Postoperative PSA Level	0.70	4.42	0.01	79.00
	%			
Nerve-Sparing Surgery (Yes)	55%	-	-	-
Unilateral Nerve-Sparing (%)	51%	-	-	-

**Table 2:** Spearman’s correlation of postoperative PSA and continuous variables.

Variable	Correlation Coefficient	*p*-Value	Comments
Preoperative PSA Level	−0.0487	0.1886	No significant correlation with postoperative PSA.
Age	−0.0086	0.8163	Minimal impact of age on postoperative PSA levels.
Tumor Length	0.1285	<0.001	Positive correlation; longer tumors linked to higher postoperative PSA.
Prostate Weight	−0.0024	0.9679	Negligible influence on postoperative PSA.

**Table 3:** Kruskal–Wallis H test results for postoperative PSA and categorical variables.

Variable	Test Statistic	*p*-Value	Comments
Preoperative Gleason Score	24.2127	4.77 × 10^−4^	Statistically significant; higher Gleason scores correlate with higher PSA.
Postoperative Gleason Score	24.7385	1.57 × 10^−4^	Strong association; aggressive tumors reflect elevated PSA levels.
Pathological Tumor Stage	45.1013	3.79 × 10^−9^	Highly significant; advanced stages (e.g., pT3b) associate with higher PSA.
Nerve-Sparing Surgery	3.8608	0.0494	Significant; differences exist between nerve-sparing and non-nerve-sparing.
Side of Nerve-Sparing	6.5022	0.0387	Statistically significant; unilateral vs. bilateral sparing impacts PSA.

**Table 4:** Mean and median postoperative PSA levels by subgroup.

Subgroup	Mean PSA (ng/mL)	Median PSA (ng/mL)	Standard Deviation (ng/mL)	Comments
**Preoperative Gleason Score**				
Gleason ≤ 6	0.12	0.05	0.10	Lower PSA reflects less aggressive disease.
Gleason = 7	0.32	0.10	0.80	Moderate elevation, typical of intermediate-risk disease.
Gleason > 7	4.50	0.70	18.20	Significant variability; suggests aggressive biology.
**Pathological Tumor Stage**				
pT2	0.09	0.05	0.06	PSA remains low for organ-confined disease.
pT3a	0.22	0.05	0.45	Moderate increase linked to extraprostatic extension.
pT3b	2.14	0.20	8.50	PSA significantly elevated, indicating higher disease burden.
**Nerve-Sparing Surgery**				
Yes	0.20	0.05	0.40	Lower PSA values reflect successful nerve preservation.
No	0.65	0.10	2.30	Higher variability suggests residual disease in some cases.
**Side of Nerve-Sparing**				
Unilateral	0.30	0.05	0.50	Comparable outcomes to bilateral sparing.
Bilateral	0.35	0.10	0.60	Slightly elevated PSA but not clinically significant.

**Table 5:** Logistic regression results.

Variable	Coefficient	Standard Error	z-Score	*p*-Value
Constant	−1.38	2.76	−0.50	0.62
Preoperative PSA Level	−0.03	0.03	−0.98	0.33
Age	−0.03	0.03	−0.92	0.36
Tumor Length	0.00	0.01	0.14	0.89
Postoperative Gleason Score	0.09	0.26	0.34	0.74
Prostate Weight	0.01	0.01	1.39	0.17
Positive Lymph Nodes	0.25	0.14	1.77	0.08
Total Lymph Nodes Examined	0.02	0.03	0.93	0.35
Nerve-Sparing Surgery	−0.01	0.49	−0.01	0.99
Side of Nerve-Sparing	0.90	0.65	1.40	0.16
Gleason Score Category = 7	−0.18	0.80	−0.23	0.82
Gleason Score Category > 7	−0.57	0.80	−0.72	0.47
Pathological Tumor Stage pT3a	0.59	0.50	1.19	0.23
Pathological Tumor Stage pT3b	1.16	0.56	2.08	0.04

## Data Availability

The original contributions presented in this study are included in the article. Further inquiries can be directed to the corresponding author.
